# Case Report: Safety and efficacy of tarlatamab for SCLC in a liver transplant recipient

**DOI:** 10.3389/fonc.2026.1896195

**Published:** 2026-08-21

**Authors:** Jonas Leonhardt, Johann Pratschke, Jürgen Behr, Jeremias Götschke, Philipp Jurmeister, Diego Kauffmann-Guerrero, Frederick Klauschen, Laura Lambrecht, Paula Mras, Christian Schneider, Blerina Resuli, Amanda Tufman

**Affiliations:** 1 Department of Medicine V, University Hospital, Ludwig Maximilians University (LMU) Munich, Munich, Germany; 2 Department of Surgery, Campus Charité Mitte and Campus Virchow-Klinikum, Charité-Universitätsmedizin, Freie Universität Berlin, Humboldt-Universität zu Berlin, Berlin Institute of Health, Berlin, Germany; 3 Comprehensive Pneumology Center Munich (CPC-M), Member of the German Center for Lung Research (DZL), Munich, Germany; 4 Bavarian Cancer Research Center (BZKF), Munich, Germany; 5 Institute of Pathology, University Hospital, Ludwig Maximilians University (LMU) Munich, Munich, Germany; 6 The German Consortium for Translational Cancer Research (DKTK), Munich Partner Site, Ludwig-Maximilian University München, Munich, Germany; 7 Department of Thoracic Surgery, University Hospital, Ludwig Maximilians University (LMU) Munich, Munich, Germany

**Keywords:** delta-like ligand 3, small cell lung cancer, solid organ transplant recipient, targeted therapy, tarlatamab

## Abstract

Tarlatamab is a bispecific T-cell engager used for the treatment of small cell lung cancer (SCLC). Patients with a history of solid organ transplantation carry an elevated risk of malignancy yet are excluded from most clinical trials. We report the first case of Tarlatamab use in a liver transplant recipient with SCLC, resulting in marked tumor response and a favorable safety profile without evidence of graft rejection.

## 1 Introduction

Tarlatamab is a novel bispecific T-cell engager labeled by the Food and Drug Administration (FDA) for the treatment of small cell lung cancer (SCLC), targeting delta-like ligand 3 (DLL3) expressed by cancer cells, as well as CD3 on human T cells. Binding DLL3 on cancer cells and CD3 on T cells leads to cytolysis of the cancer cells (1).

Tarlatamab was superior to topotecan in the DeLLphi-304 trial for second-line treatment of SCLC (2). Before European Medicines Agency (EMA) approval was granted, imported Tarlatamab had already been in use for individual patients since 2024. To avoid severe toxicity, the first cycle of Tarlatamab is administered intravenously in a step-up protocol, starting with 1 mg on day 1 and 10 mg on day 8 and day 15. Therapy is then continued with 10 mg every 2 weeks (3). Main adverse events include cytokine release syndrome (CRS), a systemic inflammatory response triggered by rapid cytokine release of immune cells, resulting in hypotension, hypoxia, and fever. CRS mostly occurs in the first days after or during the first cycle of therapy. ICANS (immune effector cell-associated neurotoxicity syndrome) is the second relevant, however rare, side effect occurring also only during the initiation phase of the treatment (4).

Patients with previous solid organ transplantation are at increased risk of cancer but are excluded from most clinical trials, particularly trials of immunotherapeutic agents due to fear of organ rejection. To date, only a single case report has described the safe use of Tarlatamab in a heart transplant recipient (5). To our knowledge, the tolerability and efficiency of Tarlatamab in a liver transplant recipient has not yet been described.

## 2 Case presentation

This case report describes the first published use of Tarlatamab in a liver transplant recipient with SCLC. A 77-year-old man was first diagnosed with stage IIB (T2aN1M0, TNM 8th Edition) SCLC in March 2024. The patient had a previous history of liver transplantation in 1997 for hepatocellular carcinoma (HCC) in the setting of chronic hepatitis B and cirrhosis.

**Figure 1:**
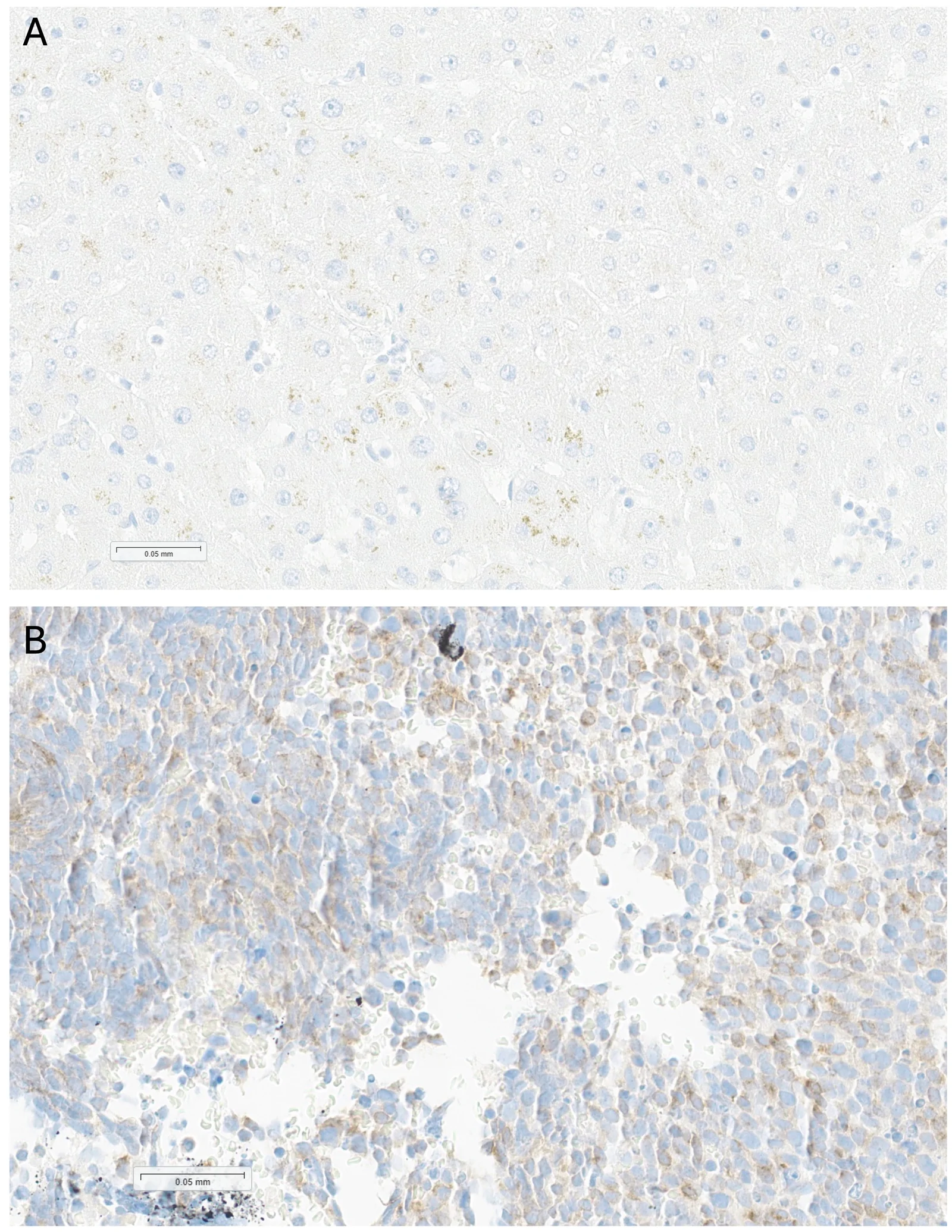
Histological sections of **(A)** graft liver, DLL3 staining, 40× – no DLL3 expression, scale bar: 0.05 mm and **(B)** primary lung tumor (SCLC), DLL3 staining, 40× – DLL3-positive, scale bar: 0,05 mm.

At first presentation, the patient had an Eastern Cooperative Oncology Group (ECOG) performance status of 0; the patient was no longer in need of immunosuppression. Treatment was initiated using definitive radiochemotherapy (51.6 Gy in 30 fractions, carboplatin AUC 5, and etoposide 100 mg/m²), five cycles, followed by surgical resection in an individual approach. Beginning 7 months after initial diagnosis, further lines of therapy were administered for disease progression including carboplatin and etoposide rechallenge, Topotecan, and palliative radiation of chest wall and brain metastases. Late-line Tarlatamab treatment was recommended by the institutional interdisciplinary tumor board. Before beginning Tarlatamab, archived FFPE (formalin-fixed, paraffin-embedded) tissue from the graft liver, resected at the time of HCC recurrence in 2023, was analyzed for DLL3 expression using immunohistochemistry. No DLL3 expression was found. This is in line with the reported absence of DLL3 expression in normal tissue (**Figure 1A**; 6). Available tumor samples of the patient’s primary lung carcinoma, however, were stained for DLL3 as well and showed positive expression (**Figure 1B**). Positive and negative controls are provided in the **Supplementary Material**.

Tarlatamab was initiated in July 2025, dosed as per FDA label beginning with 1 mg. The patient experienced a grade 2 CRS, including fever and hypotension, and transient neutropenia grade 3 was observed. Paracetamol, dexamethasone, and tocilizumab were administered; no parenteral catecholamines were required, and the patient was able to remain on the general ward. The second dose was delayed, allowing for full resolution of side effects, and administered on day 12 at 10 mg. After the second dose, the patient exhibited symptoms consistent with a grade 1 CRS, including fever and mild fatigue. We were able to administer the third dose (10 mg of Tarlatamab) on day 19 without any complications.

**Figure 2:**
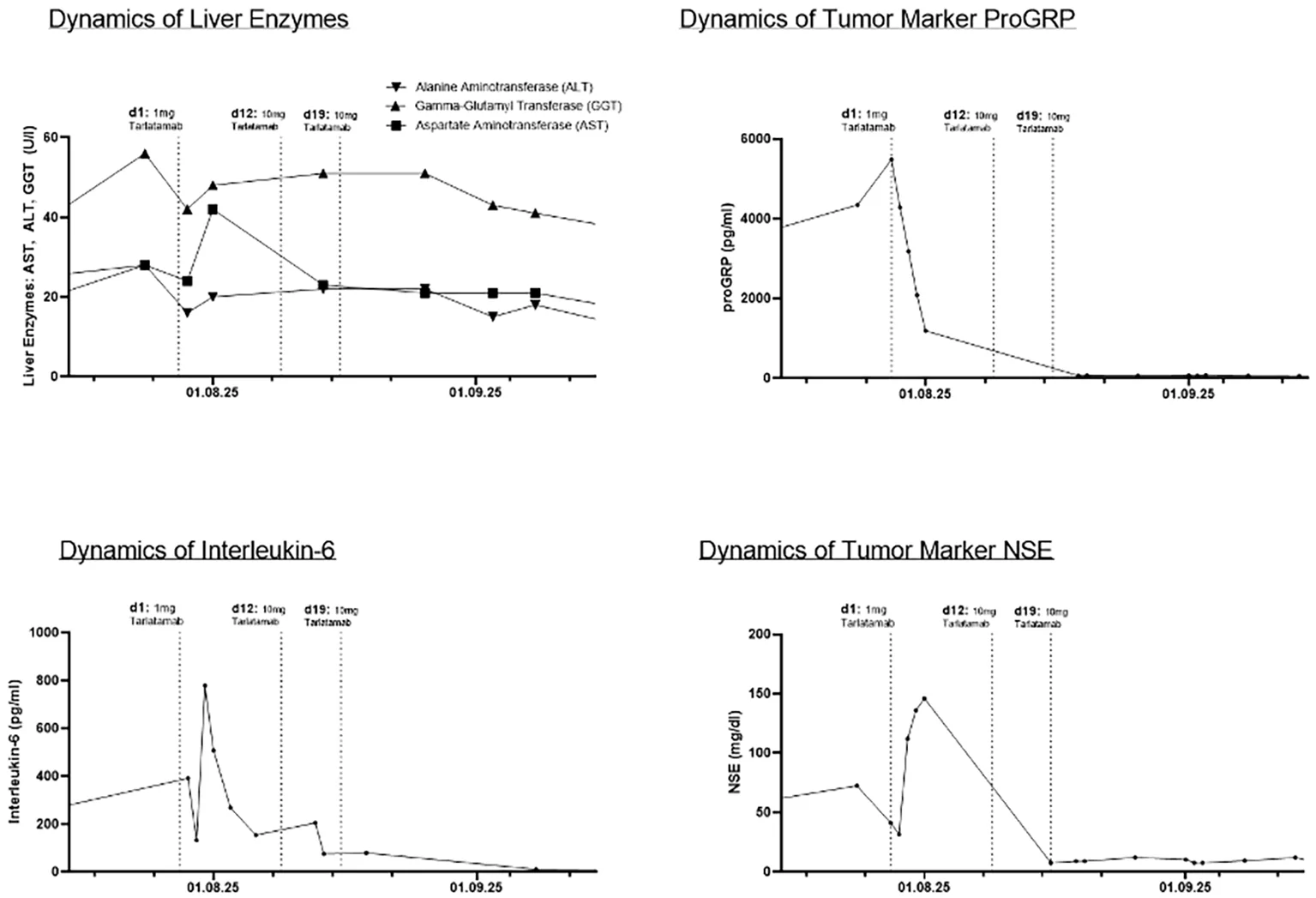
Dynamics of liver enzymes, tumor markers, ProGRP, NSE, and interleukin-6.

During the treatment, liver transaminases and liver function were closely monitored and showed no sign of early graft dysfunction (**Figure 2**). Total bilirubin remained within the normal range throughout the treatment. As the patient was on therapeutic oral anticoagulation, coagulation parameters could not be reliably used to assess hepatic synthetic function. Bile acids were not measured. Tumor markers, ProGRP, and NSE were closely monitored (**Figure 2**): ProGRP was initially 5,483 pg/mL and decreased rapidly hours after Tarlatamab administration—at 24 h, 4,283 pg/mL; 48 h, 3,179 pg/mL; 72 h, 2,083 pg/mL; 96 h, 1,187 pg/mL—and normalized by day 22 to 62.2 pg/mL. NSE rose initially from 41.2 to 146 ng/mL on day 4 and then normalized to 7.6 ng/mL by day 22. Interleukin-6 (IL-6) rose to a maximum of 779 pg/mL and fell but did not normalize over 4 weeks (45 pg/mL at day 28).

**Figure 3:**
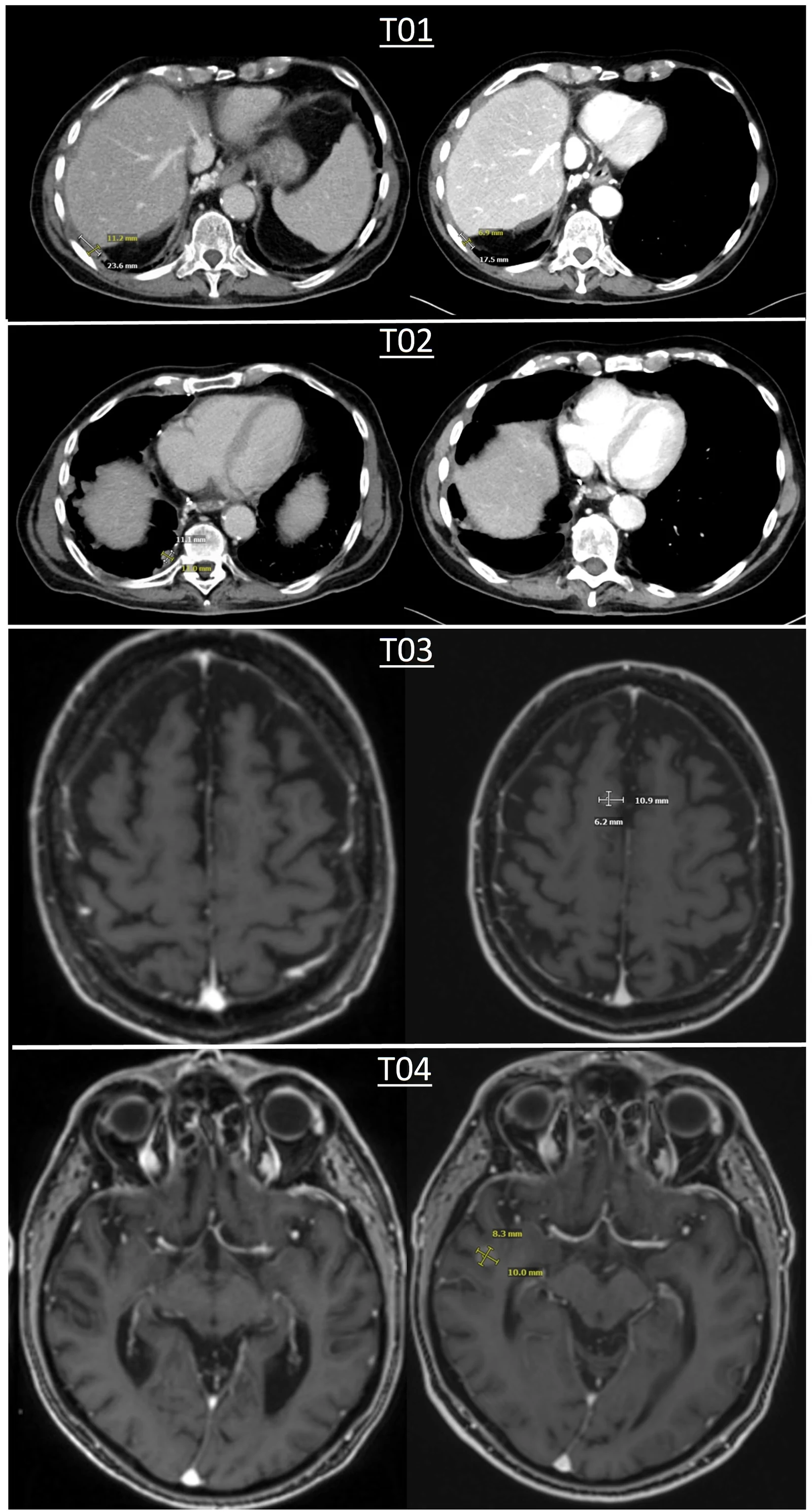
Comparison of initial staging (June 2025, left) with follow-up imaging (August 2025, right): partial response of the right paravertebral pleural lesion (T01), partial response of the right costodiaphragmatic pleural lesion (T02), and complete response of the left superior frontal gyrus metastasis (T03) and the right temporal metastasis (T04).

After three Tarlatamab infusions, chest computed tomography (CT) and brain magnetic resonance imaging (MRI) were performed (day 31) and showed partial remission of thoracic tumor sites and complete remission of cerebral metastases, as seen in **Figure 3**. The corresponding RECIST 1.1 assessment of all target lesions (T01–T04) is shown in **Table 1**.

**Table 1:** RECIST 1.1 assessment of target lesions (T01–T04) before and after Tarlatamab treatment.

Target lesion	Baseline MRI brain, 23 June 2025; PET/CT, 27 June 2025	Staging MRI brain, 27 August 2025; CT scan, 27 August 2025
T01 right paravertebral pleural lesion	11 mm	0 mm
T02 right costodiaphragmatic pleural lesion	23.6 mm	17.5 mm
T03 left superior frontal gyrus metastasis	10.9 mm	0 mm
T04 right temporal metastasis	10 mm	0 mm
Target sum	55.5 mm	17.5 mm; target response: PR

## 3 Discussion

To conclude, the presented case is the first published case documenting the tolerability and efficiency of late-line Tarlatamab for SCLC in a liver transplant recipient showing relevant efficacy, manageable safety, and no signs of organ rejection. We hypothesize that the transient increase in IL-6 and NSE followed by normalization (**Figure 2**) may reflect early cytokine release and tumor cell lysis as CRS resolved and tumor burden decreased.

Safety findings are consistent with the report of Tarlatamab in a solid organ transplant recipient by Ismail et al. In contrast to that case, our patient received Tarlatamab without concurrent immunosuppression and experienced a clear tumor response, whereas the case mentioned showed intracranial progression.

Thus, Tarlatamab may be a treatment option for SCLC in patients with previous organ transplantation. It may be relevant that graft tissue does not express DLL3. Further studies are needed to confirm the safety and efficacy of Tarlatamab in solid organ transplant recipients, as we present a single case report. Our case extends limited experience to liver transplantation. Long-term follow-up of our patient is ongoing and will be reported.

## Data Availability

The raw data supporting the conclusions of this article will be made available by the authors, without undue reservation.

## References

[1] Ahn M-J Cho BC Felip E Korantzis I Ohashi K Majem M . Tarlatamab for patients with previously treated small-cell lung cancer. N Engl J Med. (2023) 389:2063–75. doi: 10.1056/NEJMoa2307980 37861218

[2] Mountzios G Sun L Cho BC Demirci U Baka S Gümüş M . Tarlatamab in small-cell lung cancer after platinum-based chemotherapy. N Engl J Med. (2025) 393:349–61. doi: 10.1056/NEJMoa2502099 40454646

[3] Chen P-W Minocha M Kong S Jiang T Anderson ES Parkes A . Tarlatamab exposure-efficacy and exposure-safety relationships to inform dose selection in patients with small cell lung cancer. Clin Cancer Res: Off J Am Assoc For Cancer Res. (2025) 31:4688–97. doi: 10.1158/1078-0432.CCR-25-2134 40928991 PMC12616239

[4] Sands JM Champiat S Hummel H-D Paulson KG Borghaei H Alvarez JB . Practical management of adverse events in patients receiving tarlatamab, a delta-like ligand 3-targeted bispecific T-cell engager immunotherapy, for previously treated small cell lung cancer. Cancer. (2025) 131:e35738. doi: 10.1002/cncr.35738 39876075 PMC11775405

[5] Ismail A Zhang T Vickers A Boggs DH Desai A Boumber Y . Safe treatment of an extensive-stage small-cell lung cancer with tarlatamab in an orthotopic heart transplantation patient: a case report. Cureus. (2025) 17:e92622. doi: 10.7759/cureus.92622 41111642 PMC12535221

[6] Saunders LR Bankovich AJ Anderson WC Aujay MA Bheddah S Black K . A DLL3-targeted antibody-drug conjugate eradicates high-grade pulmonary neuroendocrine tumor-initiating cells *in vivo*. Sci Transl Med. (2015) 7:302ra136. doi: 10.1126/scitranslmed.aac9459 26311731 PMC4934375

